# 1-Ethyl 2-methyl 3,4-bis­(acet­yloxy)pyrrolidine-1,2-di­carboxyl­ate: crystal structure, Hirshfeld surface analysis and computational chemistry

**DOI:** 10.1107/S205698902000701X

**Published:** 2020-05-29

**Authors:** Sofia Dallasta Pedroso, Ignez Caracelli, Julio Zukerman-Schpector, Monica Soto-Monsalve, Regina H. De Almeida Santos, Carlos Roque D. Correia, Ariel L. Llanes Garcia, Huey Chong Kwong, Edward R. T. Tiekink

**Affiliations:** a Laboratório de Cristalografia, Esterodinâmica e Modelagem Molecular, Departamento de Química, Universidade Federal de São Carlos, 13565-905 São Carlos, SP, Brazil; b Departmento de Física, Universidade Federal de São Carlos, 13565-905 São Carlos, SP, Brazil; c Instituto de Química de São Carlos, Universidade de São Paulo, São Carlos, SP, Brazil; d Instituto de Química, Universidade Estadual de Campinas, UNICAMP, CP 6154, CEP 13084-917 Campinas, Brazil; e Research Centre for Crystalline Materials, School of Science and Technology, Sunway University, 47500 Bandar Sunway, Selangor Darul Ehsan, Malaysia

**Keywords:** crystal structure, pyrrolidine, Hirshfeld surface analysis, NCI plots, computational chemistry

## Abstract

The title compound, C_13_H_19_NO_8_, is based on a tetra-substituted pyrrolidine ring, which has a twisted conformation about the central C—C bond; the C_m_—C_a_—C_a_—C_me_ torsion angle is 38.26 (15)° [m = methyl­carboxyl­ate, a = acet­yloxy and me = methyl­ene]. While the N-bound ethyl­carboxyl­ate group occupies an equatorial position, the remaining substituents occupy axial positions. In the crystal, supra­molecular double-layers are formed by weak methyl- and methyl­ene-C—H⋯O(carbon­yl) inter­actions involving all four carbonyl-O atoms. The two-dimensional arrays stack along the *c* axis without directional inter­actions between them. The Hirshfeld surface is dominated by H⋯H (55.7%) and H⋯C/C⋯H (37.0%) contacts; H⋯H contacts are noted in the inter-double-layer region. The inter­action energy calculations point to the importance of the dispersion energy term in the stabilization of the crystal.

## Abstract 2

The tetra-substituted pyrrolidine ring in the title compound has a twisted conformation about the central C—C bond with the N-bound ethyl­carboxyl­ate group in an equatorial position and the remaining substituents in axial positions. In the crystal, methyl- and methyl­ene-C—H⋯O(carbon­yl) inter­actions involving all four carbonyl-O atoms lead to supra­molecular double-layers.

## Chemical context   

A number of diseases, especially diabetes but also including viral diseases, cystic fibrosis and cancer, can be treated with α-glucosidase inhibitors (Dhameja & Gupta, 2019 ▸; Kiappes *et al.*, 2018 ▸); for a review of the relevant patent literature, see Brás *et al.* (2014 ▸). Imino- and aza-sugars are strong inhibitors of the enzyme and are attracting current inter­est for chaperone therapy of Gaucher disease (Matassini *et al.*, 2020 ▸). The tri-hydroxyl-substituted compound, amino­ciclitol, (I), is a known α-glucosidase inhibitor and is a natural product, being found in several plants (Assefa *et al.*, 2020 ▸). The synthesis of (I) can proceed from several key inter­mediates (Garcia, 2008 ▸; Liu & Ma, 2017 ▸) and it is this consideration that prompted the structural investigation of the title compound, C_13_H_19_NO_8_, (II). Specifically, the HCl salt of (I) can be prepared from (II) after being subjected to a sequence of reactions comprising a reduction step, reflux acid hydrolysis, chromatographic purification on ion-exchange resin Dowex-H^+^ and, finally, hydro­chloride formation. In this way, (I)·HCl was obtained in 67% yield (Garcia, 2008 ▸). In connection with supporting structural studies (Zukerman-Schpector *et al.*, 2017 ▸) of crucial inter­mediates related to the synthesis of pharmacologically active (I), herein, the crystal and mol­ecular structures of (II) are described. This is complemented by a detailed analysis of the supra­molecular architecture by Hirshfeld surface analysis, non-covalent inter­actions plots and computational chemistry.
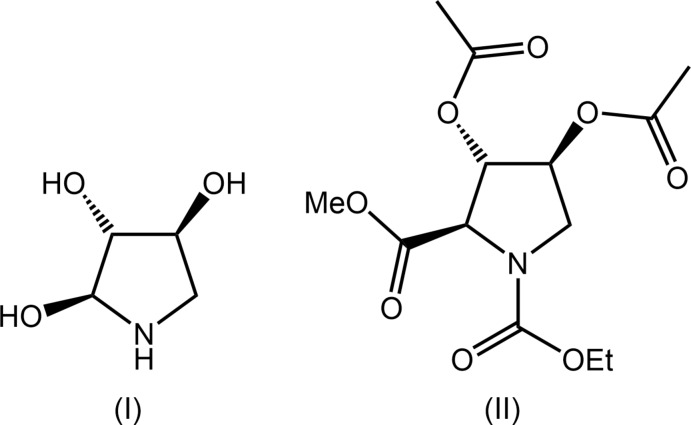


## Structural commentary   

**Figure 1:**
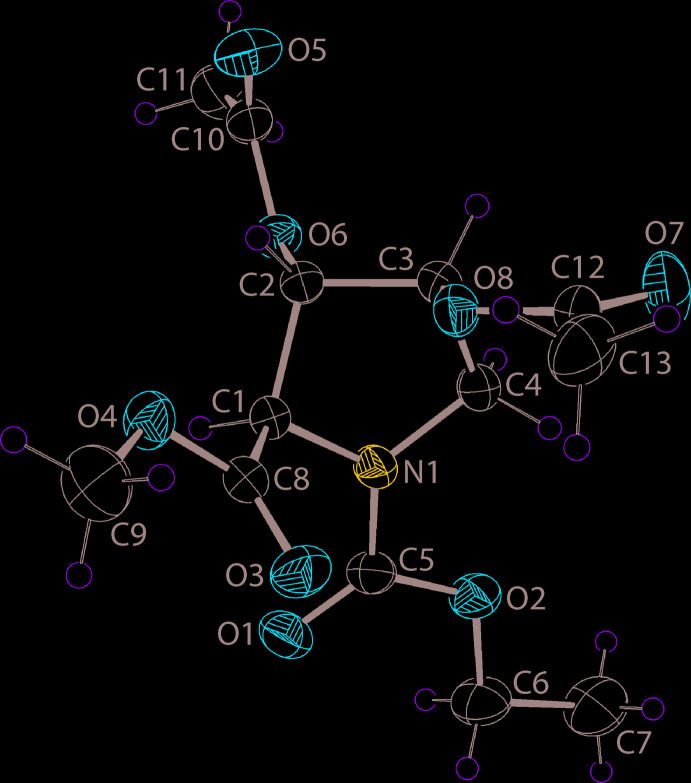
The mol­ecular structure of (II), showing the atom-labelling scheme and displacement ellipsoids at the 35% probability level.

The mol­ecular structure of (II), Fig. 1 ▸, features a tetra-substituted pyrrolidine ring. The conformation of the five-membered ring is best described as being twisted about the C2—C3 bond; the C1—C2—C3—C4 torsion angle is 38.26 (15)° indicating a (+)*syn*-clinal configuration. With respect to the five-membered ring, the *N*-bound methyl­carboxyl­ate substituent occupies an equatorial position; the sum of angles about the N1 atom amounts to 360°, indicating this is an *sp* ^2^ centre. At the C1–C3 centres, the methyl­carboxyl­ate and 2 × acet­yloxy substituents, respectively, occupy axial positions. For the mol­ecule illustrated in Fig. 1 ▸, the chirality of each of the C1–C3 atoms is *R*, *S* and *S*, respectively; the centrosymmetric unit cell contains equal numbers of each enanti­omer. When viewed towards the approximate plane through the pyrrolidine ring, the N-bound substituent is approximately co-planar, the C2-acet­yloxy lies to one side of the plane, and the C1- and C3-substituents lie to the other side.

## Supra­molecular features   

**Figure 2:**
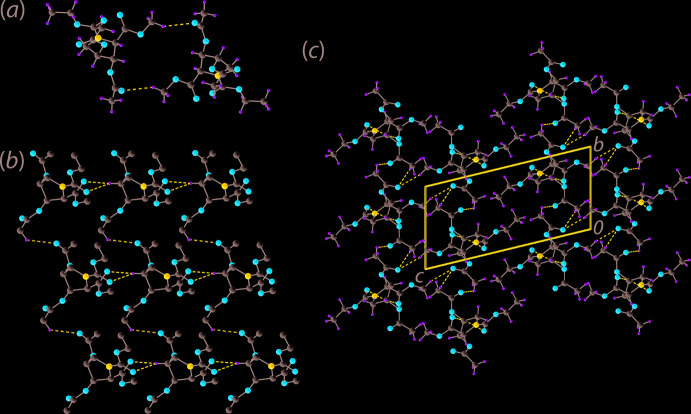
Mol­ecular packing in (II): (*a*) supra­molecular dimer sustained by methyl-C9—H⋯O5(carbon­yl) contacts, (*b*) layer sustained by methyl-C11—H⋯O7(carbon­yl) and bifurcated methyl­ene-C3—H⋯O1,O3(carbon­yl) contacts (non-participating H atoms are omitted) and (*c*) a view of the unit-cell contents shown in projection down the *a* axis. The C—H⋯O inter­actions are shown as blue dashed lines.

There are two classes of identifiable non-covalent C—H⋯O inter­actions occurring in the crystal of (II). As identified in *PLATON* (Spek, 2020 ▸), methyl-C9—H⋯O5(carbon­yl) contacts (Table 1 ▸) occur between centrosymmetrically related mol­ecules to form a dimeric aggregate and an 18-membered {⋯OCOC_3_OCH}_2_ synthon, Fig. 2 ▸(*a*). The second level, *i.e*. weaker, of C—H⋯O inter­actions assemble mol­ecules into a supra­molecular layer in the *ab* plane, Fig. 2 ▸(*b*), at separations beyond normally accepted values in *PLATON* (Spek, 2020 ▸). Here, a methyl­ene-C3—H atom is bifurcated, forming contacts with the carbonyl-O1 and O3 atoms of a translationally related mol­ecule along the *a*-axis direction. This is complemented by a methyl-C11—H⋯O7(carbon­yl) inter­action occurring along the *b-*axis direction, Fig. 2 ▸(*c*). The layer thus formed by these contacts is connected into a double-layer *via* the methyl-C9—H⋯O5(carbon­yl) inter­actions mentioned above. The double-layers stack along the *c* axis without directional inter­actions between them.

## Non-covalent inter­action plots   

Before embarking on a more detailed analysis of the overall mol­ecular packing of (II), in particular of the inter-layer region along the *c* axis, non-covalent inter­action plots (Johnson *et al.*, 2010 ▸; Contreras-García *et al.*, 2011 ▸) were calculated to analyse in more detail the nature of the specified C—H⋯O contacts described in *Supra­molecular features*. This method analyses the electron density (and derivatives) around the specified inter­molecular contacts and generates colour-based isosurfaces as detailed in the cited literature. The results, through a three-colour scheme, enable the visualization of contacts as being attractive (blue isosurface), repulsive (red) or otherwise. For the weak inter­actions in focus, a green isosurface indicates a weakly attractive inter­action.

**Figure 3:**
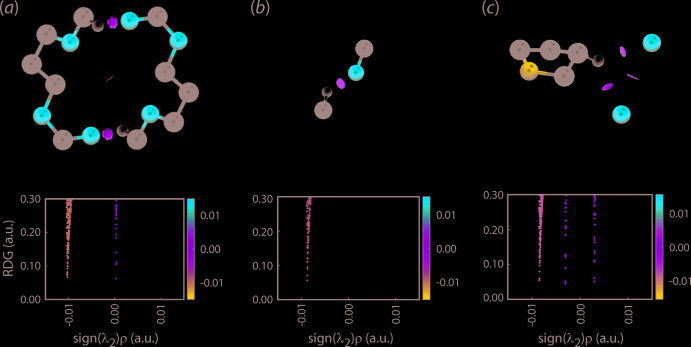
Non-covalent inter­action plots for the following inter­actions in (II): (*a*) methyl-C9—H⋯O5(carbon­yl), (b) methyl-C11—H⋯O7(carbon­yl) and (c) bifurcated methyl­ene-C3—H⋯O1,O3(carbon­yl).

The isosurfaces for three identified C—H⋯O contacts are given in the upper view of Fig. 3 ▸, and each displays a green isosurface indicating weakly attractive inter­actions. The lower views of Fig. 3 ▸ show the plots of RDG *versus* sign(λ^2^)*ρ*(*r*) for the three sets of C—H⋯O inter­actions. The green peaks apparent at density values less than 0.0 a.u. indicate these are weakly attractive inter­actions.

## Hirshfeld surface analysis   

**Figure 4:**
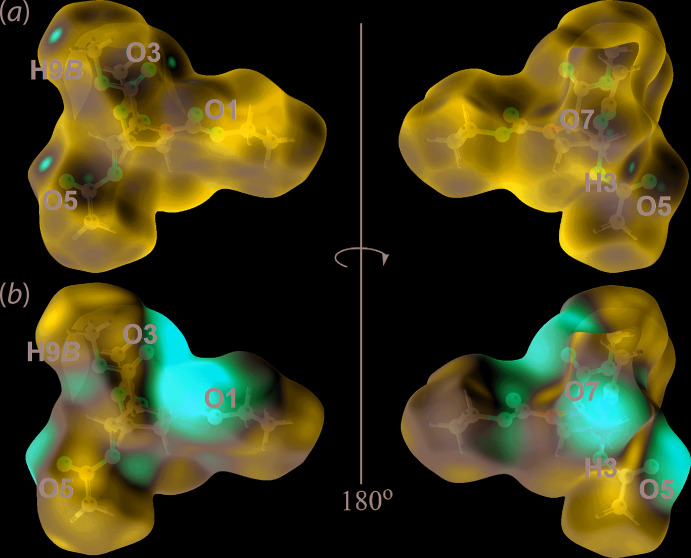
Two views of the Hirshfeld surface mapped for (II) over (*a*) *d*_norm_ in the range of −0.083 to +1.828 arbitrary units and (*b*) the calculated electrostatic potential in the range of −0.077 to 0.054 a.u.

**Figure 5:**
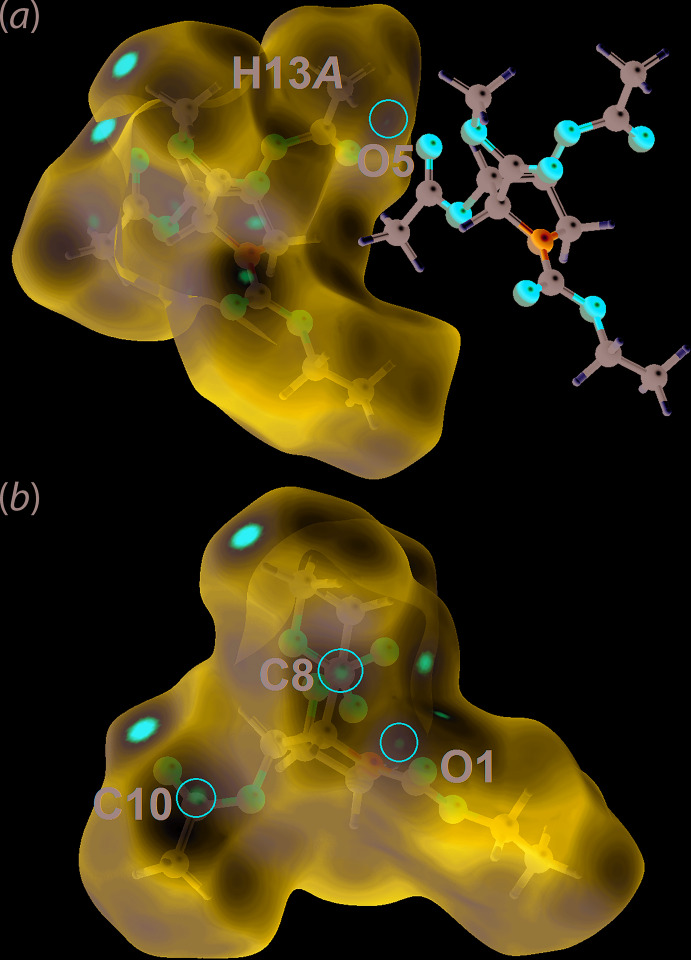
Views of the Hirshfeld surface mapped over *d*_norm_ for (II) in the range −0.083 to +1.828 arbitrary units, highlighting within red circles (*a*) a weak C—H⋯O inter­action and (*b*) C⋯O contacts.

In order to understand further the inter­actions operating in the crystal of (II), the calculated Hirshfeld surfaces were mapped over the normalized contact distance, *d*_norm_ (McKinnon *et al.*, 2004 ▸) and electrostatic potential (Spackman *et al.*, 2008 ▸) with associated two-dimensional (2-D) (full and delineated) fingerprint (FP) plots (Spackman & McKinnon, 2002 ▸). These were generated using *Crystal Explorer 17* (Turner *et al.*, 2017 ▸) following literature procedures (Tan *et al.*, 2019 ▸). The potentials were calculated using the STO-3G basis set at the Hartree–Fock level of theory. The bright-red spots on the Hirshfeld surface mapped over *d*_norm_, Fig. 4 ▸(*a*), near the carbonyl-O (O1, O3, O5 and O7) and methyl-C—H (H3 and H9*B*) atoms correspond to the C—H⋯O inter­actions listed in Table 1 ▸. These observations were confirmed through the Hirshfeld surface mapped over the calculated electrostatic potential in Fig. 4 ▸(*b*), where the surface around carbonyl-O and methyl-C—H atoms are shown in red (negative electrostatic potential) and blue (positive electrostatic potential), respectively. Besides the C—H⋯O inter­actions listed in Table 1 ▸, a long C13—H13*A*⋯O5 inter­action is reflected in the *d*_norm_ surface as a faint red spot in Fig. 5 ▸(*a*). In addition, short, intra-layer C⋯O contacts with separations 0.01–0.04 Å shorter than the sum of their van der Waals radii, Table 2 ▸, are observed as faint red spots on the *d*_norm_ surface in Fig. 5 ▸(*b*), reflecting the specific influence of the C8, C10 and O1 atoms participating in these contacts.

**Figure 6:**
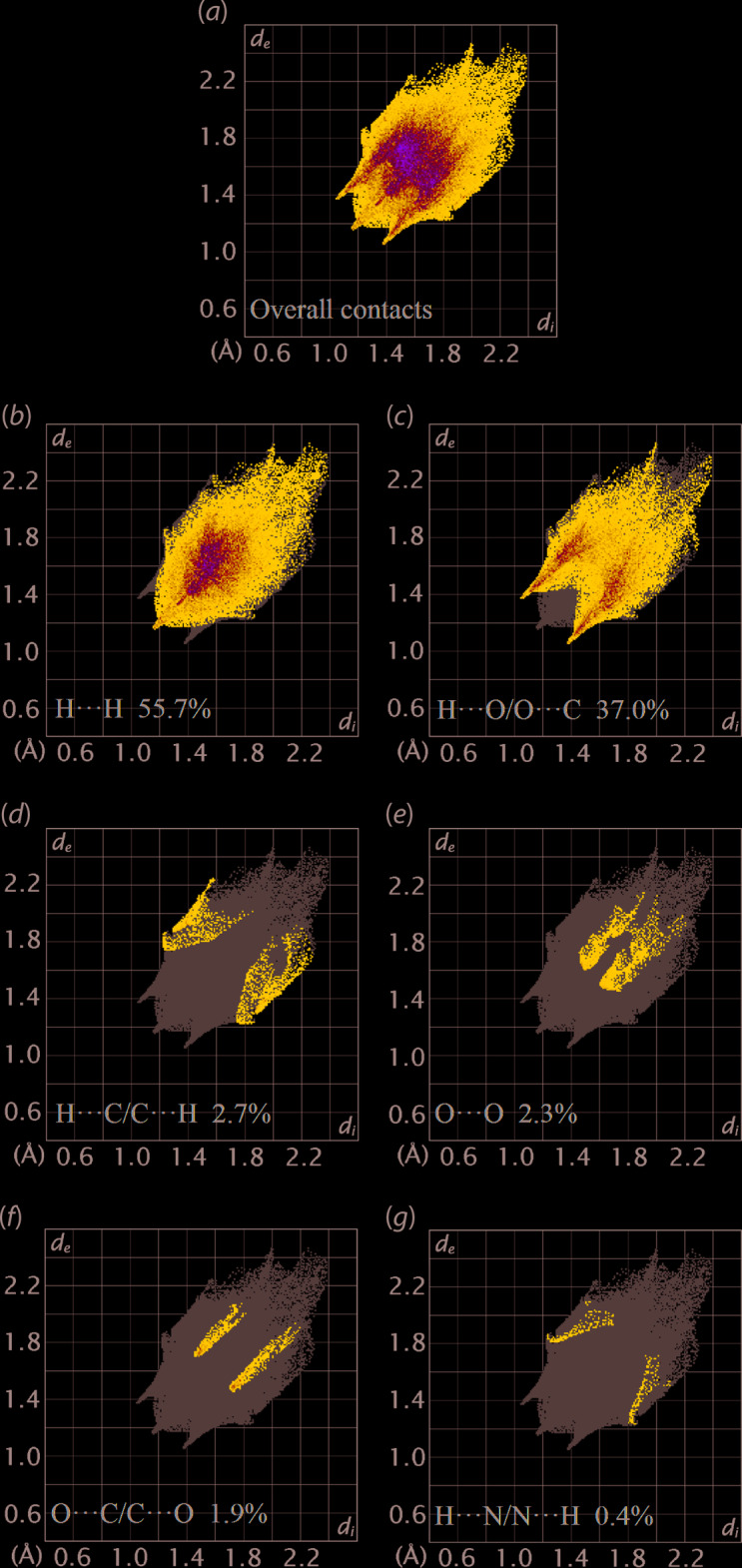
(*a*) The full two-dimensional fingerprint plot for (II) and (*b*)–(*g*) those delineated into H⋯H, O⋯H/H⋯O, C⋯H/H⋯C, O⋯O, C⋯O/O⋯C and H⋯N/N⋯H contacts, respectively.

The corresponding two-dimensional fingerprint plot for the Hirshfeld surface of (II) is shown with characteristic pseudo-symmetric wings in the upper left and lower right sides of the *d*_e_ and *d*_i_ diagonal axes, respectively, in Fig. 6 ▸(*a*). The individual H⋯H, H⋯O/O⋯H, H⋯C/C⋯H, O⋯O, O⋯C/C⋯O and H⋯N/N⋯H contacts are illustrated in the delineated two-dimensional fingerprint plots (FP) in Fig. 6 ▸(*b*)–(*g*), respectively; the percentage contributions from different inter­atomic contacts are summarized in Table 3 ▸. The H⋯H contacts contribute 55.7% to the overall Hirshfeld surface with a beak-shape distribution in the FP with shortest *d*_e_ = *d*_i_ ∼2.4 Å. This short inter­atomic H⋯H contact involving the methyl-H11*C* and methyl­ene-H4*B* atoms, Table 2 ▸, is around the sum of their van der Waals separation and occurs in the intra-layer region along the *b* axis. Consistent with the C—H⋯O inter­actions making the major contribution to the directional inter­actions in the crystal, H⋯O/O⋯H contacts contribute 37.0% to the overall Hirshfeld surface. A distinctive feature in the FP of Fig. 6 ▸(*c*), is the two symmetric spikes at *d*_e_ + *d*_i_ ∼2.4 Å. Although H⋯C/C⋯H, O⋯O, O⋯C/C⋯O and H⋯N/N⋯H appear as splash-like distributions of points at *d*_e_ + *d*_i_ ∼3.0 Å, Fig. 6 ▸(*d*)–(*g*), their contributions to the overall Hirshfeld surface are each below than 3.0%. These contacts and the remaining inter­atomic contacts have only a small effect on the packing, as the sum of their contributions to the overall Hirshfeld surface is less than 8%.

## Energy frameworks   

**Figure 7:**
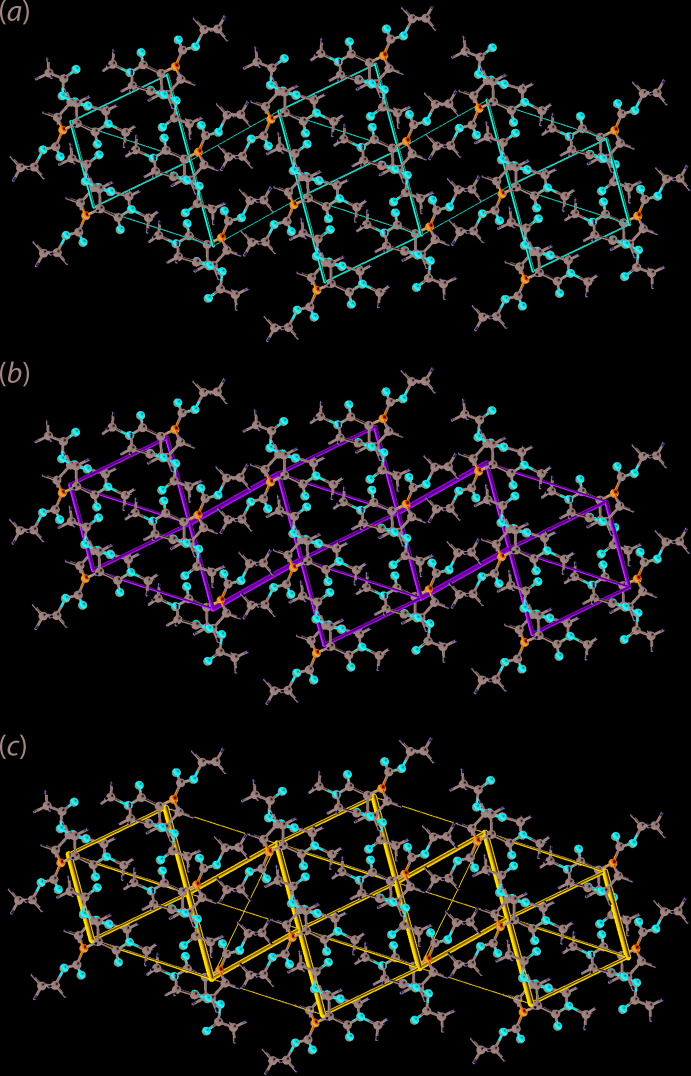
Perspective views of the energy frameworks calculated for (II) and viewed down the *b* axis showing (*a*) electrostatic potential force, (*b*) dispersion force and (*c*) total energy. The radii of the cylinders are proportional to the relative magnitudes of the corresponding energies and were adjusted to the same scale factor of 55 with a cut-off value of 5 kJ mol^−1^ within 2 × 2 × 2 unit cells.

The pairwise inter­action energies between the mol­ecules in the crystal of (II) were calculated using the wave function at the B3LYP/6-31G(d,p) level of theory. The total energy comprise four terms: electrostatic (*E*_ele_), polarization (*E*_pol_), dispersion (*E*_dis_) and exchange-repulsion (*E*_rep_) and were scaled as 1.057, 0.740, 0.871 and 0.618, respectively (Edwards *et al.*, 2017 ▸). The characteristics of the inter­molecular inter­actions in term of their energies are collated in Table 4 ▸. In the absence of conventional hydrogen bonding in the crystal, the dispersive component makes the major contribution to the inter­action energies (Table 4 ▸). According to the total inter­action energy, mol­ecules within the supra­molecular double layer are stabilized by C—H⋯O inter­action, C⋯O short contacts and long-range H⋯H contacts. Whereas mol­ecules between the supra­molecular double layers are stabilized by long-range H⋯H contacts. Views of the energy framework diagrams down the *b* axis are shown in Fig. 7 ▸ and serve to emphasize the contribution of dispersion forces in the stabil­ization of the crystal.

## Database survey   

**Figure 8:**
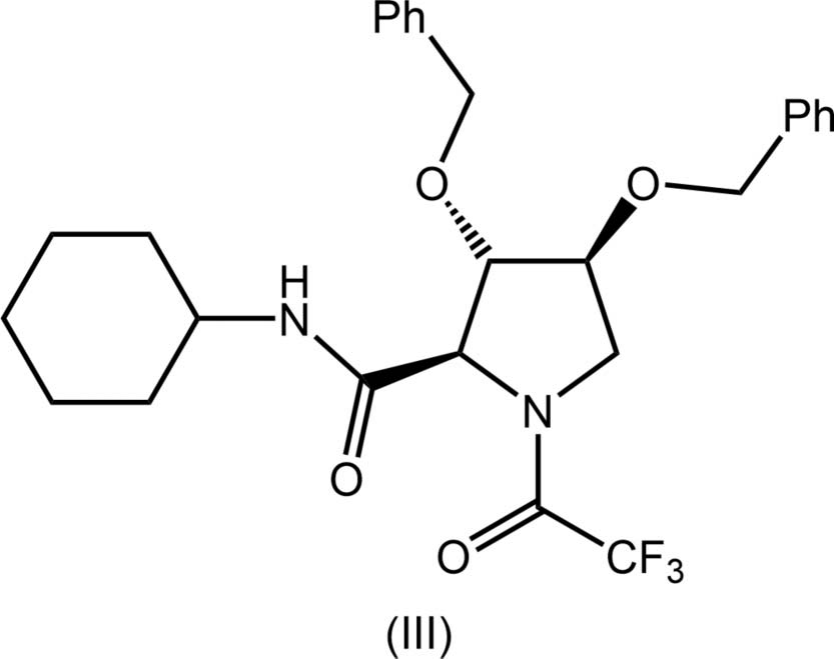
Chemical diagram for (III).

There are no close precedents for the substitution pattern observed in the tetra-substituted pyrrolidine ring of (II) with, arguably, the most closely related structure being that of (III) (KULQEP; Szcześniak *et al.*, 2015 ▸), at least in terms of the substitution pattern around the ring; the chemical diagram for (III) is shown in Fig. 8 ▸.

## Synthesis and crystallization   

A solution of (2*R*,*3S*,4*S*)-3,4-bis­(acet­yloxy)-1-(eth­oxy­carbon­yl)pyrrolidine-2-carb­oxy­lic acid (40 mg, 0.132 mmol) in methanol (1 ml) was cooled to 273–278 K after which an excess of a cold, freshly prepared solution of CH_2_N_2_ in ether was added. The mixture was stirred at room temperature for 10 min. Excess CH_2_N_2_ was eliminated by purging the balloon with a dry air flow. The purge was collected on a solution of HOAc in MeOH. The reaction solution was concentrated to dryness and the residue was purified by flash column chromatography on silica gel, using a mixture of EtOAc/*n*-hexane (1:3). Yield: 41.7 mg (qu­anti­tative) of (II). Colourless irregular crystals for the X-ray analysis were obtained by the slow evaporation of its *n*-hexane solution. M.p. 347.6–348.7 K.

The ^1^H and ^13^C NMR reflect the presence of two conformational rotamers in solution. ^1^H NMR (500 MHz, CDCl_3_): δ = 5.38 (*s*, 1H, H_3_); 5.11 (*s*, 1H, H_4_); 4.51 and 4.42 (2*s*, 1H, H_2_); 4.23–4.05 (2*m*, 2H, CH_2_CH_3_); 3.91 and 3.87 (2*dd*, *J* = 12.8 Hz and 5.5 Hz, 1H, H_4a_); 3.772 and 3.766 (2*s*, 3H, CO_2_CH_3_); 3.63 and 3.59 (2*d*, *J* = 12.8 Hz, 1H, H_4b_); 2.10 and 2.09 (2*s*, 3H, Ac); 2.01 and 2.00 (2*s*, 3H, Ac); 1.28 and 1.21 (2*t*, *J* = 7.0 Hz, 3H, CH_2_CH_3_). ^1^H NMR (500 MHz, C_6_D_6_, r.t.): δ = 5.62 (*s*, 1H, H_3_); 5.07 and 5.02 (2*ap t*, *J* = 2.7 Hz, 1H, H_3_); 4.78 (*s*, 0.6H, H_1_); 4.55 (*s*, 0.4H, H_1_); 4.12 and 4.10 (2*q*, *J* = 7.0 Hz, 0.4H, CH_2_CH_3_); 4.01–3.88 (*q* + *m*, *J* = 7.0 Hz, 2H, CH_2_CH_3_ and H_4a_); 3.84–3.76 (*m*, 1H, H_4b_); 3.65 (*dd*, *J* = 12.2 Hz and 2.4 Hz, 0.6H, H_4a_); 3.31 and 3.30 (2*s*, 3H, CO_2_CH_3_); 1.48 and 1.45 (2*s*, 3H, Ac); 1.43 and 1.42 (2*s*, 3H, Ac); 0.94 and 0.92 (2*t*, *J* = 7.0 Hz, 3H, CH_2_CH_3_). ^13^C NMR (125 MHz, CDCl_3_, r.t.): δ = 169.4; 169.3; 169.2; 168.8; 168.7; 154.8; 154.4; 77.9; 76.9; 74.5; 73.5; 63.6; 63.5; 61.9; 61.7; 52.7; 52.6; 50.6; 50.4; 20.74; 20.70; 20.65; 14.5. Microanalysis calculated for C_13_H_19_NO_8_: C 49.21, H 6.04, N 4.41%. Found: C 48.89, H 6.52, N 4.50%.

## Refinement details   

Crystal data, data collection and structure refinement details are summarized in Table 5 ▸. The carbon-bound H atoms were placed in calculated positions (C—H = 0.96–0.98 Å) and were included in the refinement in the riding-model approximation, with *U*_iso_(H) set to 1.2–1.5*U*_eq_(C).

## Supplementary Material

Crystal structure: contains datablock(s) I, global. DOI: 10.1107/S205698902000701X/hb7916sup1.cif

Structure factors: contains datablock(s) I. DOI: 10.1107/S205698902000701X/hb7916Isup2.hkl

Supporting information file. DOI: 10.1107/S205698902000701X/hb7916Isup3.cml

CCDC reference: 2005478

Additional supporting information:  crystallographic information; 3D view; checkCIF report

## Figures and Tables

**Table 1:** Hydrogen-bond geometry (Å, °)

*D*—H⋯*A*	*D*—H	H⋯*A*	*D*⋯*A*	*D*—H⋯*A*
C9—H9*B*⋯O5^i^	0.96	2.53	3.403 (3)	151
C3—H3⋯O1^ii^	0.98	2.62	3.419 (2)	139
C3—H3⋯O3^ii^	0.98	2.61	3.453 (2)	144
C11—H11*A*⋯O7^iii^	0.96	2.66	3.329 (3)	127

Symmetry codes: (i) 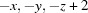; (ii) 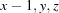; (iii) 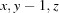.

**Table 2:** Summary of short inter­atomic contacts (Å) in (I)^*a*^

Contact	Distance	Symmetry operation
H4*B*⋯H11*C*	2.32	*x* + 1, *y* + 1, *z*
H9*B*⋯O5^*b*^	2.42	−*x*, −*y*, −*z* + 2
H3⋯O1^*b*^	2.55	*x* − 1, *y*, *z*
H3⋯O3 ^*b*^	2.53	*x* − 1, *y*, *z*
H11*A*⋯O7^*b*^	2.59	−*x*, −*y* + 1, −*z*
H13*A*⋯O5	2.58	*x* + 1, *y* + 1, *z*
C8⋯O5	3.191 (2)	*x* + 1, *y*, *z*
C10⋯O1	3.204 (2)	*x* − 1, *y*, *z*
C10⋯O7	3.185 (3)	*x*, *y* − 1, *z*

Notes: (*a*) The inter­atomic distances are calculated in *Crystal Explorer 17* (Turner *et al.*, 2017 ▸) whereby the *X*—H bond lengths are adjusted to their neutron values. (*b*) These inter­actions correspond to the inter­action listed in Table 1 ▸.

**Table 3:** Percentage contributions of inter­atomic contacts to the Hirshfeld surface for (II)

Contact	Percentage contribution
H⋯H	55.7
H⋯O/O⋯H	37.0
H⋯C/C⋯H	2.7
O⋯O	2.3
O⋯C/C⋯O	1.9
H⋯C/C⋯H	0.4

**Table 4:** Summary of inter­action energies (kJ mol^−1^) calculated for (II)

Contact	*R* (Å)	*E*_ele_	*E*_pol_	*E*_dis_	*E*_rep_	*E*_tot_
Intra-double-layer						
C3—H3⋯O1^ii^ +						
C3—H3⋯O3^ii^ +						
O1⋯C10^iv^ +						
O5⋯C8^ii^	6.8	−19.4	−8.3	−33.6	19.3	−44.0
H9*B*⋯H13*C* ^v^ +						
H13*B*⋯H13*B* ^v^	8.2	−5.1	−1.6	−28.4	11.0	−24.5
C13—H13*A*⋯O5^vi^ +						
H4*B*⋯H11*C* ^vi^ +						
H7*C*⋯H11*B* ^vi^	9.0	−8.8	−2.1	−20.8	10.7	−22.4
C11—H11*A*⋯O7^iii^ +						
C13—H13*C*⋯O4^iii^ +						
C10⋯O7^vii^	7.9	−8.1	−2.9	−20.2	12.5	−20.6
H9*A*⋯H13*A* ^viii^ +						
H9*C*⋯H9*C* ^viii^	9.3	−6.5	−2.1	−19.8	14.4	−16.7
C9—H9*B*⋯O5^i^	9.1	−10.2	−2.3	−12.9	13.9	−15.1
C7—H7*B*⋯O7^ix^	9.9	−3.6	−0.9	−15.1	4.8	−14.7
Inter-double-layer region						
H4*A*⋯H6*A* ^*x*^ +						
H7*B*⋯H11*B* ^*x*^	8.1	−5.0	−1.8	−41.4	17.3	−31.9
H7*A*⋯H11*C* ^xi^	8.9	−0.9	−0.4	−10.8	6.2	−6.8

Symmetry codes: (i) −*x*, −*y*, −*z* + 2; (ii) *x* − 1, *y*, *z*; (iii) *x*, *y* − 1, *z*; (iv) *x* + 1, *y*, *z*; (v) −*x*, −*y* + 1, −*z* + 2; (vi) *x* + 1, *y* + 1, *z*; (vii) *x*, *y* + 1, *z*; (viii) −*x* + 1, −*y* + 1, −*z* + 2; (ix) −*x*, −*y* + 1, −*z*; (*x*) −*x*, −*y*, −*z* + 1; (xi) −*x* − 1, −*y*, −*z* + 1.

**Table 5:** Experimental details

Crystal data	
Chemical formula	C_13_H_19_NO_8_
*M*_r_	317.29
Crystal system, space group	Triclinic, *P* 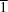
Temperature (K)	290
*a*, *b*, *c* (Å)	6.8291 (5), 7.8670 (11), 15.814 (3)
α, β, γ (°)	100.607 (11), 99.011 (10), 105.054 (7)
*V* (Å^3^)	787.5 (2)
*Z*	2
Radiation type	Mo *K*α
μ (mm^−1^)	0.11
Crystal size (mm)	0.40 × 0.35 × 0.10

Data collection	
Diffractometer	Enraf Nonius TurboCAD-4
No. of measured, independent and observed [*I* > 2σ(*I*)] reflections	4880, 4573, 2571
*R*_int_	0.020
(sin θ/λ)_max_ (Å^−1^)	0.703

Refinement	
*R*[*F* ^2^ > 2σ(*F* ^2^)], *wR*(*F* ^2^), *S*	0.042, 0.128, 0.99
No. of reflections	4573
No. of parameters	203
H-atom treatment	H-atom parameters constrained
Δρ_max_, Δρ_min_ (e Å^−3^)	0.18, −0.17

Computer programs: *CAD-4 EXPRESS* (Enraf Nonius, 1989 ▸), *XCAD4* (Harms & Wocadlo, 1995 ▸), *SIR2014* (Burla *et al.*, 2015 ▸), *SHELXL2018/3* (Sheldrick, 2015 ▸), *ORTEP-3 for Windows* (Farrugia, 2012 ▸), *MarvinSketch* (ChemAxon, 2010 ▸), *DIAMOND* (Brandenburg, 2006 ▸) and *publCIF* (Westrip, 2010 ▸).

## supplementary crystallographic information

### Crystal data

**Table:**

C_13_H_19_NO_8_	*Z* = 2
*M**_r_* = 317.29	*F*(000) = 336
Triclinic, *P*1	*D*_x_ = 1.338 Mg m^−^^3^
*a* = 6.8291 (5) Å	Mo *K*α radiation, λ = 0.71073 Å
*b* = 7.8670 (11) Å	Cell parameters from 25 reflections
*c* = 15.814 (3) Å	θ = 10.8–18.2°
α = 100.607 (11)°	µ = 0.11 mm^−^^1^
β = 99.011 (10)°	*T* = 290 K
γ = 105.054 (7)°	Irregular, colourless
*V* = 787.5 (2) Å^3^	0.40 × 0.35 × 0.10 mm

### Data collection

**Table:**

Enraf Nonius TurboCAD-4 diffractometer	θ_max_ = 30.0°, θ_min_ = 2.7°
Radiation source: Enraf Nonius FR590	*h* = −9→9
non–profiled ω/2θ scans	*k* = 0→11
4880 measured reflections	*l* = −22→21
4573 independent reflections	3 standard reflections every 120 min
2571 reflections with *I* > 2σ(*I*)	intensity decay: 2%
*R*_int_ = 0.020	

### Refinement

**Table:**

Refinement on *F*^2^	Primary atom site location: structure-invariant direct methods
Least-squares matrix: full	Secondary atom site location: difference Fourier map
*R*[*F*^2^ > 2σ(*F*^2^)] = 0.042	Hydrogen site location: inferred from neighbouring sites
*wR*(*F*^2^) = 0.128	H-atom parameters constrained
*S* = 0.99	*w* = 1/[σ^2^(*F*_o_^2^) + (0.0579*P*)^2^ + 0.0763*P*] where *P* = (*F*_o_^2^ + 2*F*_c_^2^)/3
4573 reflections	(Δ/σ)_max_ = 0.001
203 parameters	Δρ_max_ = 0.18 e Å^−^^3^
0 restraints	Δρ_min_ = −0.17 e Å^−^^3^

### Special details

**Table:**

Geometry. All esds (except the esd in the dihedral angle between two l.s. planes) are estimated using the full covariance matrix. The cell esds are taken into account individually in the estimation of esds in distances, angles and torsion angles; correlations between esds in cell parameters are only used when they are defined by crystal symmetry. An approximate (isotropic) treatment of cell esds is used for estimating esds involving l.s. planes.

### Fractional atomic coordinates and isotropic or equivalent isotropic displacement parameters (Å^2^)

**Table:**

	*x*	*y*	*z*	*U*_iso_*/*U*_eq_	
O1	0.28490 (19)	0.08131 (19)	0.64555 (9)	0.0568 (3)	
O2	0.12213 (18)	0.24723 (18)	0.57354 (8)	0.0513 (3)	
O3	0.35221 (19)	0.33818 (18)	0.83767 (9)	0.0586 (3)	
O4	0.20434 (18)	0.14935 (17)	0.91551 (8)	0.0504 (3)	
O5	−0.5275 (2)	−0.0729 (2)	0.83254 (9)	0.0662 (4)	
O6	−0.32790 (16)	−0.06328 (15)	0.73314 (7)	0.0424 (3)	
O7	−0.2014 (3)	0.6135 (2)	0.78612 (11)	0.0753 (4)	
O8	−0.08692 (17)	0.40628 (15)	0.84074 (7)	0.0426 (3)	
N1	0.0250 (2)	0.1764 (2)	0.69258 (9)	0.0433 (3)	
C1	0.0322 (2)	0.0990 (2)	0.76913 (10)	0.0372 (3)	
H1	0.041065	−0.024702	0.752792	0.045*	
C2	−0.1782 (2)	0.0948 (2)	0.79177 (10)	0.0357 (3)	
H2	−0.177363	0.096071	0.853908	0.043*	
C3	−0.2178 (2)	0.2626 (2)	0.76694 (10)	0.0388 (3)	
H3	−0.364464	0.257939	0.758613	0.047*	
C4	−0.1353 (2)	0.2683 (2)	0.68313 (11)	0.0430 (4)	
H4A	−0.244190	0.204920	0.631224	0.052*	
H4B	−0.076679	0.392201	0.679209	0.052*	
C5	0.1565 (2)	0.1615 (2)	0.63798 (10)	0.0424 (4)	
C6	0.2518 (3)	0.2410 (3)	0.50973 (13)	0.0631 (5)	
H6A	0.234590	0.116529	0.480776	0.076*	
H6B	0.396809	0.298206	0.538377	0.076*	
C7	0.1853 (4)	0.3399 (4)	0.44469 (15)	0.0818 (7)	
H7A	0.041201	0.282919	0.417435	0.123*	
H7B	0.266749	0.337723	0.400460	0.123*	
H7C	0.204764	0.463198	0.474089	0.123*	
C8	0.2155 (2)	0.2132 (2)	0.84352 (11)	0.0404 (3)	
C9	0.3834 (3)	0.2283 (4)	0.98760 (14)	0.0766 (7)	
H9A	0.506912	0.224946	0.966399	0.115*	
H9B	0.370682	0.160845	1.032257	0.115*	
H9C	0.391528	0.351722	1.012025	0.115*	
C10	−0.4972 (2)	−0.1374 (2)	0.76304 (12)	0.0461 (4)	
C11	−0.6347 (3)	−0.3050 (3)	0.69971 (16)	0.0697 (6)	
H11A	−0.581462	−0.404559	0.706145	0.104*	
H11B	−0.639848	−0.289168	0.640732	0.104*	
H11C	−0.771979	−0.329842	0.711270	0.104*	
C12	−0.0926 (3)	0.5768 (2)	0.84243 (13)	0.0500 (4)	
C13	0.0585 (4)	0.7066 (3)	0.92107 (15)	0.0696 (6)	
H13A	0.194942	0.735689	0.908739	0.104*	
H13B	0.058933	0.652183	0.970653	0.104*	
H13C	0.018502	0.815216	0.934292	0.104*	

### Atomic displacement parameters (Å^2^)

**Table:**

	*U*^11^	*U*^22^	*U*^33^	*U*^12^	*U*^13^	*U*^23^
O1	0.0482 (7)	0.0740 (9)	0.0610 (8)	0.0303 (7)	0.0206 (6)	0.0223 (7)
O2	0.0509 (7)	0.0668 (8)	0.0457 (6)	0.0205 (6)	0.0212 (5)	0.0231 (6)
O3	0.0431 (6)	0.0550 (8)	0.0714 (8)	0.0012 (6)	0.0106 (6)	0.0205 (7)
O4	0.0476 (6)	0.0545 (7)	0.0462 (6)	0.0117 (6)	0.0003 (5)	0.0186 (6)
O5	0.0573 (8)	0.0702 (10)	0.0703 (9)	0.0073 (7)	0.0301 (7)	0.0171 (8)
O6	0.0357 (5)	0.0405 (6)	0.0475 (6)	0.0065 (5)	0.0088 (5)	0.0092 (5)
O7	0.0854 (11)	0.0571 (9)	0.0929 (11)	0.0370 (8)	0.0070 (9)	0.0284 (8)
O8	0.0440 (6)	0.0367 (6)	0.0497 (6)	0.0151 (5)	0.0096 (5)	0.0122 (5)
N1	0.0395 (7)	0.0576 (9)	0.0448 (7)	0.0230 (6)	0.0144 (6)	0.0243 (6)
C1	0.0345 (7)	0.0403 (8)	0.0423 (8)	0.0148 (6)	0.0098 (6)	0.0168 (7)
C2	0.0327 (7)	0.0348 (8)	0.0397 (8)	0.0087 (6)	0.0082 (6)	0.0105 (6)
C3	0.0322 (7)	0.0404 (8)	0.0458 (8)	0.0125 (6)	0.0082 (6)	0.0127 (7)
C4	0.0411 (8)	0.0497 (10)	0.0446 (8)	0.0186 (7)	0.0094 (7)	0.0196 (7)
C5	0.0359 (7)	0.0520 (10)	0.0389 (8)	0.0111 (7)	0.0086 (6)	0.0118 (7)
C6	0.0635 (12)	0.0808 (15)	0.0527 (11)	0.0219 (11)	0.0292 (9)	0.0195 (10)
C7	0.0946 (17)	0.112 (2)	0.0566 (12)	0.0356 (15)	0.0357 (12)	0.0387 (13)
C8	0.0357 (7)	0.0424 (9)	0.0477 (9)	0.0155 (7)	0.0101 (6)	0.0154 (7)
C9	0.0645 (13)	0.0948 (18)	0.0570 (12)	0.0172 (12)	−0.0142 (10)	0.0169 (12)
C10	0.0358 (8)	0.0443 (9)	0.0596 (10)	0.0088 (7)	0.0106 (7)	0.0200 (8)
C11	0.0448 (10)	0.0558 (12)	0.0917 (16)	−0.0003 (9)	0.0043 (10)	0.0075 (11)
C12	0.0516 (9)	0.0402 (9)	0.0677 (12)	0.0181 (8)	0.0229 (9)	0.0213 (9)
C13	0.0803 (14)	0.0425 (11)	0.0764 (14)	0.0050 (10)	0.0192 (12)	0.0075 (10)

### Geometric parameters (Å, º)

**Table:**

O1—C5	1.2098 (19)	C3—H3	0.9800
O2—C5	1.345 (2)	C4—H4A	0.9700
O2—C6	1.446 (2)	C4—H4B	0.9700
O3—C8	1.1961 (19)	C6—C7	1.484 (3)
O4—C8	1.3312 (19)	C6—H6A	0.9700
O4—C9	1.445 (2)	C6—H6B	0.9700
O5—C10	1.195 (2)	C7—H7A	0.9600
O6—C10	1.3499 (19)	C7—H7B	0.9600
O6—C2	1.4385 (18)	C7—H7C	0.9600
O7—C12	1.195 (2)	C9—H9A	0.9600
O8—C12	1.348 (2)	C9—H9B	0.9600
O8—C3	1.4496 (19)	C9—H9C	0.9600
N1—C5	1.351 (2)	C10—C11	1.483 (3)
N1—C1	1.4518 (19)	C11—H11A	0.9600
N1—C4	1.464 (2)	C11—H11B	0.9600
C1—C8	1.524 (2)	C11—H11C	0.9600
C1—C2	1.528 (2)	C12—C13	1.496 (3)
C1—H1	0.9800	C13—H13A	0.9600
C2—C3	1.521 (2)	C13—H13B	0.9600
C2—H2	0.9800	C13—H13C	0.9600
C3—C4	1.523 (2)		
			
C5—O2—C6	115.92 (14)	O2—C6—H6B	110.4
C8—O4—C9	115.73 (15)	C7—C6—H6B	110.4
C10—O6—C2	116.20 (12)	H6A—C6—H6B	108.6
C12—O8—C3	118.19 (13)	C6—C7—H7A	109.5
C5—N1—C1	121.34 (13)	C6—C7—H7B	109.5
C5—N1—C4	125.77 (13)	H7A—C7—H7B	109.5
C1—N1—C4	112.89 (12)	C6—C7—H7C	109.5
N1—C1—C8	111.47 (13)	H7A—C7—H7C	109.5
N1—C1—C2	102.32 (12)	H7B—C7—H7C	109.5
C8—C1—C2	113.23 (13)	O3—C8—O4	124.90 (16)
N1—C1—H1	109.9	O3—C8—C1	125.34 (15)
C8—C1—H1	109.9	O4—C8—C1	109.73 (13)
C2—C1—H1	109.9	O4—C9—H9A	109.5
O6—C2—C3	108.78 (12)	O4—C9—H9B	109.5
O6—C2—C1	106.68 (12)	H9A—C9—H9B	109.5
C3—C2—C1	103.23 (12)	O4—C9—H9C	109.5
O6—C2—H2	112.5	H9A—C9—H9C	109.5
C3—C2—H2	112.5	H9B—C9—H9C	109.5
C1—C2—H2	112.5	O5—C10—O6	122.72 (16)
O8—C3—C2	102.26 (11)	O5—C10—C11	125.62 (16)
O8—C3—C4	111.80 (12)	O6—C10—C11	111.66 (16)
C2—C3—C4	103.54 (13)	C10—C11—H11A	109.5
O8—C3—H3	112.8	C10—C11—H11B	109.5
C2—C3—H3	112.8	H11A—C11—H11B	109.5
C4—C3—H3	112.8	C10—C11—H11C	109.5
N1—C4—C3	103.35 (12)	H11A—C11—H11C	109.5
N1—C4—H4A	111.1	H11B—C11—H11C	109.5
C3—C4—H4A	111.1	O7—C12—O8	122.72 (18)
N1—C4—H4B	111.1	O7—C12—C13	126.78 (18)
C3—C4—H4B	111.1	O8—C12—C13	110.46 (16)
H4A—C4—H4B	109.1	C12—C13—H13A	109.5
O1—C5—O2	125.04 (15)	C12—C13—H13B	109.5
O1—C5—N1	124.93 (15)	H13A—C13—H13B	109.5
O2—C5—N1	110.02 (14)	C12—C13—H13C	109.5
O2—C6—C7	106.76 (17)	H13A—C13—H13C	109.5
O2—C6—H6A	110.4	H13B—C13—H13C	109.5
C7—C6—H6A	110.4		
			
C5—N1—C1—C8	76.02 (19)	C2—C3—C4—N1	−27.36 (15)
C4—N1—C1—C8	−103.88 (15)	C6—O2—C5—O1	−0.3 (3)
C5—N1—C1—C2	−162.67 (14)	C6—O2—C5—N1	−179.62 (15)
C4—N1—C1—C2	17.43 (18)	C1—N1—C5—O1	2.0 (3)
C10—O6—C2—C3	−96.72 (15)	C4—N1—C5—O1	−178.13 (17)
C10—O6—C2—C1	152.54 (13)	C1—N1—C5—O2	−178.67 (14)
N1—C1—C2—O6	80.73 (14)	C4—N1—C5—O2	1.2 (2)
C8—C1—C2—O6	−159.17 (12)	C5—O2—C6—C7	178.98 (17)
N1—C1—C2—C3	−33.83 (15)	C9—O4—C8—O3	−6.4 (3)
C8—C1—C2—C3	86.27 (14)	C9—O4—C8—C1	171.57 (15)
C12—O8—C3—C2	−176.59 (13)	N1—C1—C8—O3	−8.7 (2)
C12—O8—C3—C4	73.23 (17)	C2—C1—C8—O3	−123.40 (18)
O6—C2—C3—O8	168.91 (11)	N1—C1—C8—O4	173.33 (13)
C1—C2—C3—O8	−78.04 (13)	C2—C1—C8—O4	58.60 (17)
O6—C2—C3—C4	−74.78 (14)	C2—O6—C10—O5	3.0 (2)
C1—C2—C3—C4	38.26 (15)	C2—O6—C10—C11	−176.72 (15)
C5—N1—C4—C3	−173.74 (15)	C3—O8—C12—O7	0.4 (2)
C1—N1—C4—C3	6.15 (18)	C3—O8—C12—C13	−177.58 (14)
O8—C3—C4—N1	82.01 (15)		

### Hydrogen-bond geometry (Å, º)

**Table:**

*D*—H···*A*	*D*—H	H···*A*	*D*···*A*	*D*—H···*A*
C9—H9*B*···O5^i^	0.96	2.53	3.403 (3)	151
C3—H3···O1^ii^	0.98	2.62	3.419 (2)	139
C3—H3···O3^ii^	0.98	2.61	3.453 (2)	144
C11—H11*A*···O7^iii^	0.96	2.66	3.329 (3)	127

Symmetry codes: (i) −*x*, −*y*, −*z*+2; (ii) *x*−1, *y*, *z*; (iii) *x*, *y*−1, *z*.
